# Treatment by Suggestion without Sleep

**Published:** 1907-07-27

**Authors:** Douglas Bryan

**Affiliations:** Spa House, Leicester


### Editor's Letter-Box.

[Our Correspondents are reminded that prolixity is a great bar to publication, and that brevity of style and conciseness of statement greatly facilitate early insertion.]

### "TREATMENT BY SUGGESTION WITHOUT SLEEP."

Sir,—In your journal of July 6 I see Dr. Ash, in his paper on "Treatment by Suggestion without Sleep," makes this remarkable statement, "that hypnotic treatment necessitates a complete surrendering of faculties to the operator, with a period of unconsciousness for the patient." Now this is quite incorrect, and it is by statements such as these, made by medical men, that we get the "great aversion to its use."

I consider that the word "unconsciousness" is quite inadmissible as used in the foregoing statement, for it conveys to the medical and lay mind that a state is induced in which there is inability to cognise impressions, from within or without, which are ordinarily capable of producing physical or mental sensations. Compare the state of the man rendered unconscious by a blow on the head and the man hypnotised: in the first case the man lies like a log, absolutely unresponsive to stimuli, with most likely some alteration in pulse, respiration, pupils, and facial aspect; on the other hand, in the hypnotised there is a state induced in which there is ability to cognise impressions from within or without, to reason and carry on an intelligent conversation; the person is more or less under the control of the operator, and at any time can be immediately dehypnotised. I grant that in both conditions there may be no recollection of what has taken place during the unconscious state and that induced by hypnotism, but the similarity between the two conditions is hardly comparable, for in the unconscious state the amnesia is real and complete, but in the hypnotic state it is artificial and very frequently incomplete. To go further into the matter is quite unnecessary, as anyone with ordinary powers of observation would immediately recognise the great differences between the two states.

There is another portion of the same paragraph with which I entirely disagree—it is that in which Dr. Ash says "hypnotic treatment necessitates a complete surrendering of faculties to the operator." To make such an assertion as this is calculated to do immense harm to the propagation of treatment by hypnotic suggestion, in that it suggests the possibility of all manner of crimes being committed on or by the hypnotised person, and would naturally prevent many people from adopting a line of treatment which might be their only hope of cure. The observations and experiments of those who have made hypnotism and hypnotic phenomena their especial study all tend to show that, while the operator has a great control over the hypnotised person, it is by no means as complete as Dr. Ash would have us believe, even during the deep somnambulic states; and I think we should take the opinions of such men before we, who have had little experience as compared with theirs, exploit our own. I will not trespass further on your valuable space, although there are several more debatable points in the paper, but in conclusion would like to say that the making of inaccurate statements with regard to a subject like hypnotism—which is looked upon with so much suspicion both by members of the medical profession and the general public—is much to be deplored, especially when coming from a medical man who has previously written on the subject, and who has evidently had a little experience in hypnotic phenomena; for though this form of treatment is gradually making progress all over the country, yet there is much prejudice to be overcome. Still, if it is remembered that there is no case on record in which hypnotic treatment has produced any bad result when used by an experienced medical hypnotist, we who use it in our practices have a very strong argument with which to combat our opponents, and they are usually men who know practically nothing about the subject, but pick up unguarded statements such as Dr. Ash has made; for I feel convinced that he cannot substantiate his remarks except by taking some extremely exceptional case and making that the standpoint for his sweeping assertions.

Yours faithfully,

Douglas Bryan, M.R.C.S., L.R.C.P.

Spa House, Leicester, July 21.

